# Can HbA1c Alone Be Safely Used to Guide Insulin Therapy in African Youth with Type 1 Diabetes?

**DOI:** 10.1155/2023/1179830

**Published:** 2023-04-24

**Authors:** Thereza Piloya-Were, Lucy W. Mungai, Antoinette Moran, Lauren M. Yauch, Nicholas Christakis, Lin Zhang, Robert McCarter, Stuart Chalew

**Affiliations:** ^1^Department of Pediatrics, Makerere University College of Health Sciences, Kampala, Uganda; ^2^Department of Pediatrics, University of Nairobi, Nairobi, Kenya; ^3^Division of Pediatric Endocrinology and Diabetes, Department of Pediatrics, School of Medicine, University of Minnesota, Minneapolis, MN, USA; ^4^School of Medicine, Louisiana State University Health Sciences Center, New Orleans, LA, USA; ^5^Division of Biostatistics, School of Public Heath, University of Minnesota, Minneapolis, MN, USA; ^6^Biostatistics and Epidemiology (retired), Children's National Medical Center and the George Washington University, Washington, DC, USA; ^7^Division of Pediatric Endocrinology and Diabetes, Department of Pediatrics, School of Medicine, Louisiana State University Health Sciences Center, New Orleans, LA, USA; ^8^Children's Hospital of New Orleans, New Orleans, LA, USA

## Abstract

**Introduction:**

The relationship of HbA1c versus the mean blood glucose (MBG) is an important guide for diabetes management but may differ between ethnic groups. In Africa, the patient's glucose information is limited or unavailable and the management is largely guided by HbA1c. We sought to determine if the reference data derived from the non-African populations led to an appropriate estimation of MBG from HbA1c for the East African patients.

**Methods:**

We examined the relationship of HbA1c versus MBG obtained by the continuous glucose monitoring in a group of East African youth having type 1 diabetes in Kenya and Uganda (*n* = 54) compared with the data obtained from A1c-derived average glucose (ADAG) and glucose management indicator (GMI) studies. A self-identified White (European heritage) population of youth (*n* = 89) with type 1 diabetes, 3–18 years old, living in New Orleans, LA, USA metropolitan area (NOLA), was studied using CGM as an additional reference.

**Results:**

The regression equation for the African cohort was MBG (mg/dL) = 32.0 + 16.73 × HbA1c (%), *r* = 0.55, *p* < 0.0001. In general, the use of the non-African references considerably overestimated MBG from HbA1c for the East African population. For example, an HbA1c = 9% (74.9 mmol/mol) corresponded to an MBG = 183 mg/dL (10.1 mmol/L) in the East African group, but 212 mg/dL (11.7 mmol/L) using ADAG, 237 mg/dL (13.1 mmol/L) using GMI and 249 mg/dL (13.8 mmol/L) using NOLA reference. The reported occurrence of serious hypoglycemia among the African patients in the year prior to the study was 21%. A reference table of HbA1c versus MBG from the East African patients was generated.

**Conclusions:**

The use of non-African-derived reference data to estimate MBG from HbA1c generally led to the overestimation of MBG in the East African patients. This may put the East African and other African patients at higher risk for hypoglycemia when the management is primarily based on achieving target HbA1c in the absence of the corresponding glucose data.

## 1. Introduction

HbA1c has often been considered as an important surrogate for mean blood glucose (MBG) in the assessment of diabetes management [1]. However, many factors have been recognized, which can alter the level of HbA1c independently of glucose level [2, 3]. Identification of these factors is important for the accurate clinical interpretation of an individual patient's glycemic control, prediction of complications, and clinical decisions regarding changes in insulin dosing.

Of particular note is the finding of higher HbA1c levels at any given level of glucose in American individuals of African heritage (AH) compared to individuals of non-Hispanic, European White background (W). Depending on the methodology used for the assay of HbA1c and determination of blood glucose level, HbA1c has been reported to range from 0.2 to 2 percent units higher after the adjustment for MBG in AH compared to W populations [4–8]. Simply put, HbA1c may overestimate the actual MBG in a large number of AH patients compared to W patients.

These observations may have particular relevance for diabetes management as hypoglycemia has been reported to occur with greater frequency in non-White patients despite their having higher HbA1c levels [9–11]. The occurrence of hypoglycemia may be particularly troublesome when a treatment to the HbA1c target is the primary management approach [12]. This seems to be a paradoxical observation as in the Diabetes Control and Complications Trial (DCCT) and other studies of the occurrence of hypoglycemia where lower HbA1c has been associated with more hypoglycemia [13, 14].

Guidelines have been published for the use of glycemic metrics in clinical practice for diabetes management in children [15–18]. In less-resourced areas of the world such as Africa, the use of self-monitored capillary glucose is prohibitively expensive and a point of care clinic HbA1c may be the main and sometimes the only metric for the assessment of glycemic control and for guiding insulin dosing [16]. However, if HbA1c overestimates the MBG of youth with diabetes living in Africa, as it does for African diaspora populations, then treatment to HbA1c target might lead to inadvertent over prescription of insulin and more frequent occurrence of hypoglycemia [14]. The purpose of this data analysis was to compare the population data for the relationship between HbA1c and MBG derived from a relatively homogenous population of youth in Uganda and Kenya, East Africa, with similar data gathered from the previously cited populations outside of Africa and comprised of large numbers of non-AH patients.

## 2. Methodology

### 2.1. Sources of Data

Data were chosen from populations where the mean blood glucose had been determined from the continuous glucose monitoring (CGM) paired with an appropriately timed corresponding HbA1c and the regression relationship between the two variables calculated.

### 2.2. East Africa

HbA1c and MBG were collected as part of a prior study examining the use of CGM in youth with T1D from patients in Uganda and Kenya, East Africa. Details of the study goals, population, and data collection were previously reported [19]. Briefly, MBG was obtained using a Freestyle Libre Pro CGM system. Patients were blinded to the CGM output. Point of care HbA1c was measured using a Hemocue HbA1c 501 System (Angelholm, Sweden; percent coefficient of variation 1.7–5.5%) in Uganda and a Siemens DCA Vantage System (Frimley, UK; percent CV 2.5%) in Kenya. The upper limit for both assays was 14% (130 mmol/mol). Both HbA1c assay systems were National Glycohemoglobin Standardization Program (NGSP) certified and traceable to International Federation of Clinical Chemistry and Laboratory Medicine (IFCC) reference materials and methods. Data were included for analysis from nonpregnant patients without acute illness, 2–26 yrs old, having type 1 diabetes for at least 6 months, HbA1c <14% and duration of CGM monitoring for at least 7 days. Patients with HbA1cs ≥ 14 were excluded for concern of distorting the relationship of HbA1c with MBG. Of the 68 patients previously studied, 14 were excluded due to sensor use of less than a week and/or HbA1c ≥ 14.

The relationship between MBG and HbA1c was found to be similar for the two clinical centers in Kenya and Uganda, and the patient data were combined for analysis and presentation. The goal of clinical management of patients in the East African clinics was to achieve HbA1c target of <7% (53 mmol/mol). The occurrence of severe hypoglycemia (loss of consciousness) during the year prior to starting the CGM trial was reported by the patients [19].

### 2.3. New Orleans

A self-identified White (European heritage) population of youth *n* = 89, with type 1 diabetes, 3–18 years old, living in the New Orleans metropolitan area (NOLA) was studied [20]. MBG from these patients was derived from Dexcom CGM system data extracted from a Clarity™ for the four weeks before the clinic visit. HbA1c was drawn by venipuncture at the time of the clinic visit and measured by immunoassay in the clinical lab of the Children's Hospital of New Orleans. This assay is aligned with the NGSP. The regression equation determined from MBG and HbA1c pairs for this “NOLA” population was MBG (mg/dL) = −131.1 + 42.2 × HbA1c(%) (Conversion formula MBG (mmol/L) = −2.24 + 0.214 *∗* HbA1c(mmol/mol)).

The regression equation from the population used to develop the Glucose Management Indicator (GMI) [21] was chosen as a comparison as it represented a large population of patients with paired MBG and HbA1c. This population information has been proposed as a guide to the relationship between MBG derived from CGM and HbA1c by its authors. MBG was derived from Dexcom CGM equipment. This population was predominantly White European heritage. MBG regressed on HbA1c was calculated from data in Table 1 of reference [21]. MBG (mg/dL) = −137.5 + 41.66 × HbA1c (%) (Conversion formula MBG (mmol/l) = −2.7 + 0.212 × HbA1c (mmol/mol)).

A1c-derived average glucose (ADAG) study data have been an often used reference for patients and providers for interpreting the relationship between HbA1c and MBG [18, 22]. Data were collected from 11 centers in the US, Europe, Africa, and Asia. Participants were aged 18–70 years. White patients comprised about 74% of the ADAG participants overall [22]. Medtronic MiniMed equipment was used for CGM in this study. HbA1c was the average value from four different NGSP-approved assays. The regression equation from the ADAG study was MBG (mg/dL) = −46.7 + 28.7 × HbA1c(%) [22] (Conversion formula MBG (mmol/L) = 0.582 + 0.152 × HbA1c(mmol/mol)).

### 2.4. Analysis and Comparison of Data

In the East African population, two regression analyses were conducted. One was a simple linear regression and the second was a Bayesian regression model with heteroscedasticity fitted by using JAGS software that follows the method used for the table in the ADAG study [22]. The estimated Bayesian model is MBG = 27.85 + 17.11 *∗* HbA1c (%), with Var(MBG|HbA1c) = 3074 *∗* HbA1c^0.04^. The 95% confidence intervals of MBG for different levels of HbA1c in Table 1 are based on the fitted Bayesian model to facilitate the comparison with the previously published data from the ADAG study [18, 22].

The simple regression plots were generated from each population and overlaid together in a single figure to facilitate the visual comparison of differences between the East African patients and comparison populations at integral values for HbA1c.

We have previously used the Hemoglobin Glycation Index (HGI) to assess for interindividual and between-group variation/bias in HbA1c not due to blood glucose levels [23, 24]. HGI = observed HbA1c−predicted HbA1c from the population regression equation. The Glucose Management Index (GMI) is a proposed estimate for predicted HbA1c. GMI = 3.31 + 0.02392 X mean blood glucose [21]. Using the mean blood glucose from CGM for the East African patients, we can calculate GMI and then HGI = observed HbA1c−GMI.

Conversion of HbA1c % to IFCC mmol/mol = 10.929 *∗* (HbA1c% − 2.15), MBG (mg/dL) to SI units of mmol/l = MBG (mg/dL) *∗* 0.0555.

## 3. Results

**Figure 1:**
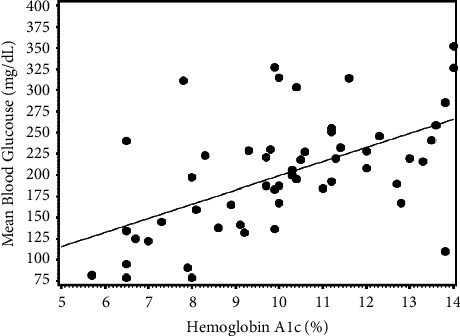
Scatterplot of mean blood glucose (mg/dL) by CGM for at least 7 days versus HbA1c for East African patients with type 1 diabetes *n* = 54. Simple regression equation is MBG (mg/dL) = 32.0 + 16.73 *∗* HbA1c(%), *r* = 0.55, *p* < 0.0001. To convert HbA1c % to IFCC mmol/mol = 10.929 *∗* (HbA1c% − 2.15), MBG (mg/dL) to SI units of mmol/l = MBG (mg/dL) *∗* 0.0555.

The regression relationship of MBG on HbA1c was determined from pediatric and young adult patients with type 1 diabetes from clinics in Kenya and Uganda, *n* = 54. The average age was 17.0 ± 5.1 (range 4–26) years, 43% female, and the average diabetes duration was 7.6 ± 4.7 years. Mean HbA1c = 10.1 ± 2.3% (86.9 mmol/mol) and MBG = 201 ± 66 mg/dL (11.2 ± 3.7 mmol/L). The scatterplot for these patients is shown in Figure 1. The simple linear regression equation derived for this population was MBG (mg/dL) = 32.00 + 16.73 × HbA1c (%) Pearson correlation *r* = 0.55, *p* < 0.0001, (MBG (mmol/L) = 3.77 + 0.085 × HbA1c (mmol/mol)). There was no influence of age, gender or clinical center on MBG in a multiple-variable model including HbA1c.

**Figure 2:**
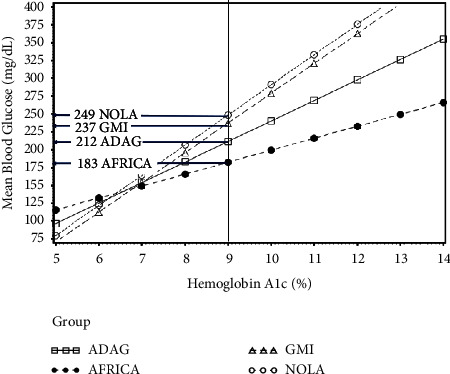
Comparison of regression equation plots for a population of African youth (AFRICA) compared with data from the GMI and ADAG study populations and a pediatric population from New Orleans (NOLA). Using data from the other reference populations would overpredict MBG for the African youth. While an HbA1c level of 9% (79.9 mmol/mol) corresponded to an MBG level of 183 mg/dL (10.2 mmol/L) in data from African youth, this level of HbA1c would be interpreted as 212 mg/dL (11.7 mmol/L) based on ADAG data, 237 mg/dL (13.1 mmol/L) from the GMI relationship and 249 mg/dL (13.8 mmol/L) using NOLA data. To convert HbA1c % to IFCC mmol/mol = 10.929 *∗* (HbA1c% − 2.15), MBG (mg/dL) to SI units of mmol/l = MBG (mg/dL) *∗* 0.0555.

The population regression relationships for MBG on HbA1c are shown in Figure 2 for the East African group along with the other populations such as non-Hispanic White youth in New Orleans (NOLA), as well as the GMI and ADAG study populations. At levels of HbA1c over 6%, the East African patients had the lowest average MBG. For example, in the East African patients, HbA1c = 8% (63.9 mmol/mol) corresponded to MBG = 166 mg/dL (9.2 mmol/L) in that population. However, HbA1c = 8% (63.9 mmol/mol) corresponded to 183 mg/dL (10.1 mmol/L) with the ADAG reference, MBG = 196 mg/dL (10.8 mmol/L) in the GMI population, and 206 mg/dL (11.4 mmol/L) from NOLA. Similarly, an HbA1c = 9% corresponded to MBG = 183 mg/dL (10.1 mmol/L) in the African group but corresponded to 212 mg/dL (11.7 mmol/L) with ADAG, 237 mg/dL (13.1 mmol/L) with GMI and 249 mg/dL (13.8 mmol/L) with NOLA. The divergence between African and the other populations increased with higher values of HbA1c.

Hemoglobin Glycation Index (HGI) using Glucose Management Index (GMI): another way to assess differences between populations is to calculate HGI. We calculated HGI as described in the Methods section above from GMI. If the East African patients had been taken from a random sampling of the population from which the GMI equation was derived, then the mean HGI should have been approximately zero. However, the mean HGI = 2.00 with a standard deviation of 1.92. Thus, on average, the HbA1c of East African patients was 2 HbA1c percent units higher than expected from the GMI population. The calculated *t*-value for this difference was 7.64 (*p* < 0.0001) indicating that the mean HGI was considerably different from zero not due to chance.

Table 1 is a reference guide for the estimation of MBG from HbA1c in the East African patients along with 95% confidence interval values.

The reported occurrence of severe hypoglycemia in the African patients in the year prior to the CGM study was 21% [19].

## 4. Discussion

Understanding the relationship between HbA1c and MBG is important for the clinical management of patients with diabetes. To our knowledge, the current data assessment is the first to compare the relationship between HbA1c and MBG derived from CGM in young Black African patients with type 1 diabetes with other reference populations. The East African patients are a relatively genetically homogenous group whereas the reference populations are primarily from the US and contained large numbers of patients of non-Hispanic White European heritage (W). Even African heritage (AH) populations and other groups in the US may be genetically heterogenous. We found that for HbA1c levels over 7% (53 mmol/mol), CGM MBG was considerably lower at any given level of HbA1c for the African patients compared to the other populations. It has been previously reported that AH populations living in the US also have lower MBG at any given level of HbA1c than W patients [4–7]. Our current study suggests that reliance on widely used reference standards such as ADAG and GMI for the relationship of HbA1c vs MBG would lead to overestimation of actual MBG for the African patients. Data from the current study do not allow us to propose a mechanism for the observed disparity of HbA1c between ethnic groups. However, others have discussed potential mechanism/s for this observation though none have been established conclusively [2, 3, 25].

We previously hypothesized that a lack of recognition of the lower estimated MBG among AH vs W populations might lead clinicians to inadvertently prescribe more insulin to AH patients with T1D in order to bring glycemic control into a recommended HbA1c target range. This potentially could lead to greater occurrence of hypoglycemia in AH patients [26]. Indeed, evidence for greater occurrence of hypoglycemia in AH patients has been reported from the U.S. In the Type 1 Diabetes Exchange study even though HbA1c was highest in AH patients, 16% of AH patients reported having one or more severe episodes of hypoglycemia in the past year as compared with 7% of W patients and 6% of Hispanic patients [9]. Similarly, in the Pediatric Diabetes Consortium from the U.S., where AH patients also had the highest HbA1c of all ethnic groups, 11% of AH patients reported having one or more severe episodes of hypoglycemia compared to 4% of W patients and 7% of Hispanic patients [10]. Among the African youth in the present study, 21% reported having had a severe hypoglycemic episode causing impairment of consciousness in the year before their trial of CGM [19]. In this East African population, access to analogue insulins and home glucose monitoring is often prohibitively expensive, and management guided largely by a goal of getting patients to an HbA1c target of 7% or lower. Potentially, African patients may be exposed to more aggressive insulin therapy in an effort to reach the HbA1c target if clinicians are using the more familiar ADAG and GMI population references. Furthermore, East African patients may not have the availability of insulin analogues which are thought to help reduce glycemic variability and risk of hypoglycemia [27]. In clinical situations where insulin management is based on patient-generated home glucose data, and HbA1c is not available at the time of clinic visit, AH patients did not have the occurrence of hypoglycemia higher than W patients [14].

The strengths of this study are that the relationship between HbA1c and MBG was determined for a young relatively homogeneous Black East African population where population-based reference data is lacking. The study demonstrates for the first time how the use of non-African reference data for HbA1c vs MBG would lead to overestimation of MBG for East African patients. The use of CGM-derived MBG data precluded potential bias that might occur when intermittent capillary glucose sampling is used as the source for MBG. A number of limitations should be considered. Our sample of African patients is relatively small and localized to East Africa which potentially may limit its generalizability to other regions of Africa and the wider African diaspora. Different HbA1c assays and CGM systems were employed in the various studies compared. There were also differences in the duration of CGM monitoring. The Freestyle Libre Pro system was used in the East African cohort, while Dexcom systems were used for the GMI and NOLA populations and Medtronic MiniMed equipment for the ADAG study. However, bias in the Libre system is not likely to be the source of the consistently lower MBG reading in African patients, as accuracy studies for FDA labelling as well as other clinical comparisons of sensors did not demonstrate a bias for lower glucose levels for the Libre sensor [28–30]. Furthermore, the finding of lower MBG levels at any given level of HbA1c for AH populations compared to W populations has been a robust finding across different populations and locales, different HbA1c assays and different approaches to glucose ascertainment and duration of glucose monitoring [4–8, 31]. Thus, the difference between the East African patients with the other populations is likely real although the magnitude of the difference might change if a standardized approach to HbA1c assay and CGM had been used across the different populations and locales.

Medical conditions and socioeconomic factors prevalent in East Africa known to influence HbA1c levels independently of MBG were not identified and excluded in the African patients. Sickle trait and G6PD deficiency would have had the tendency to raise MBG relative to HbA1c [32]. While iron, folate, and B12 deficiencies potentially would lower observed MBG relative to HbA1c [33–35]. The prevalence of these various conditions in our study patient population might contribute to the between-population differences in the relationship of HbA1c versus MBG. However, regardless of the mechanism for differences in the relationship of MBG versus HbA1c, clinicians need to be aware of these differences and potential impact on medical decision-making. The occurrence of hypoglycemia over the prior year in this study was retrospective by patient/parent report and specific details of individual patient management over the prior year were not available for correlation.

In the absence of reliable glucose data, caution is advisable in African patients when using HbA1c as a guide to insulin therapy especially when the treatment goal is the achievement of an HbA1c target of ≤7%. We offer Table 1 derived from the East African population as an initial reference guide for clinicians to consider when actual glucose data from African patients is limited or unavailable. It should be kept in mind that besides differences in the relationship of HbA1c and MBG between ethnic groups, there are also consistent differences between individuals in a given population [23, 24]. Thus, when the availability of ongoing blood glucose monitoring is limited, one clinical approach might be to use limited resources available to get some intensively sampled capillary BGs, or CGM data on one or more occasions from individual patients to pair with their concurrent HbA1c. This would enable clinicians to better individually define the HbA1c versus MBG relationship for specific patients and apply this information to future occasions when blood glucose levels are unavailable and HbA1c is the only metric of glycemic control. We believe that increased awareness that HbA1c is associated with lower MBG in African patients compared to other ethnicities may help reduce the occurrence of severe hypoglycemia. Additional data collection will be necessary to access the generalizability of Table 1 for other regions of Africa and the potential influence of factors such as sickle trait, G6PD, as well as iron, folate and B12 deficiencies on the relationship of MBG and HbA1c. Additional information will help guide approaches to management that will be most effective in improving glycemic control without the undue occurrence of severe hypoglycemia especially in high-risk populations with limited resources.

## Figures and Tables

**Table 1:** Guide to the relationship of HbA1c with mean blood glucose (MBG) and 95% confidence interval (CI) range for African youth with type 1 diabetes.

HbA1c (%)	HbA1c (mmol/mol)	MBG (mg/dL)	MBG (mmol/l)	MBG 95% CI (mg/dL)	MBG 95% CI (mmol/l)
5	31.1	116	6.4	98–129	5.4–7.2
6	42.1	132	7.3	115–146	6.4–8.1
7	53.0	149	8.3	132–163	7.3–9.1
8	63.9	166	9.2	149–181	8.3–10
9	74.9	183	10.1	166–198	9.2–11
10	85.8	199	11.1	183–215	10–12
11	96.7	216	12.0	200–232	11–13
12	107.7	233	12.9	217–249	12–14
13	118.6	250	13.8	234–266	13–15
14	129.5	266	14.8	251–283	14–16

## Data Availability

The data of the African patients used in this study are available upon request. Data of populations outside of Africa used for reference were previously collected and cited in the paper.

## Abbreviations

CGM: Continuous glucose monitoring
T1D: Type 1 diabetes
W: Non-Hispanic White, European heritage
AH: African heritage
ADAG: A1c-derived average glucose
GMI: Glucose management indicator
NOLA: New Orleans, Louisiana
NGSP: National Glycohemoglobin Standardization Program
IFCC: International Federation of Clinical Chemistry and Laboratory Medicine.
